# Prosocial behavior associated with trait mindfulness, psychological capital and moral identity among medical students: a moderated mediation model

**DOI:** 10.3389/fpsyg.2024.1431861

**Published:** 2024-11-20

**Authors:** Nana Liu, Yanjun Cao, Haibo Xu

**Affiliations:** ^1^School of Nursing, Xuzhou Medical University, Xuzhou, China; ^2^Department of Neurology, The Second Hospital, Cheeloo College of Medicine, Shandong University, Jinan, China; ^3^School of Management, Xuzhou Medical University, Xuzhou, China; ^4^Center for Mental Health Education and Research, Xuzhou Medical University, Xuzhou, China; ^5^Research Center for Psychological Crisis Prevention and Intervention of College Students in Jiangsu Province, Xuzhou Medical University, Xuzhou, China

**Keywords:** prosocial behavior, trait mindfulness, psychological capital, moral identity, medical students, moderated mediation model

## Abstract

**Purpose:**

As future doctors, medical students’ prosocial behaviors may affect the relationship between doctors and patients. This study aims to explore the effects of trait mindfulness on prosocial behaviors, as well as the mediating role of psychological capital and the moderating role of moral identity among medical students.

**Methods:**

A cross-sectional survey was conducted between July and October 2023 across four medical colleges in China, using cluster random sampling. The questionnaire included general demographic information, the Prosocial Tendencies Measurement Scale, the Five-Facet Mindfulness Questionnaire, the Psychological Capital Questionnaire, and the Moral Identity Scale. The SPSS 25.0 and PROCESS v3.4 macro were used for descriptive statistics, correlation analysis, and mediation and moderation analyses.

**Results:**

A total of 2,285 samples were included. The analyses showed that prosocial behavior was positively correlated with trait mindfulness, psychological capital, and moral identity (*r* = 0.293, 0.444, and 0.528, *p* < 0.01); trait mindfulness predicts prosocial behavior (*β* = 0.292, 95% CI [0.253, 0.332]); and psychological capital played a partial mediation role between trait mindfulness and prosocial behaviors (*β* = 0.413, 95% CI [0.368, 0.459]). Furthermore, moral identity played the moderating roles between trait mindfulness and prosocial behavior (*β* = 0.049, 95% CI [0.011, 0.087]) and between PsyCap and prosocial behavior (*β* = 0.062, 95% CI [0.032, 0.092]).

**Conclusion:**

Trait mindfulness, psychological capital, and moral identity are conducive to the development of medical students’ prosocial behavior. These findings provide evidence for the cultivation of prosocial behaviors and for the development of mental health courses, which should be tailored to medical students.

## Introduction

During the COVID-19 outbreak, several countries mobilized medical students as volunteers to participate in voluntary rescue activities to help patients relieve their illness and suffering (Chawowska et al., 2021; Chermside-Scabbo et al., 2021). For instance, in Poland, students were tasked with taking patients’ temperature or and gathering medical histories face to face (Chawowska et al., 2021). In the United States, students were assigned work in outpatient clinics to assist patients in transitioning to institutional telehealth platforms, thereby minimizing in-person visits (Rupley et al., 2020). Although a majority of medical students express their willingness to engage, actual participation in such activities remains relatively rare (Byrne et al., 2023). This phenomenon raises critical questions regarding the intrinsic motivations that drive such prosocial behavior. As future doctors, medical students’ prosocial behaviors may affect the relationship between doctors and patients. What are the underlying forces that inspire medical students to engage in acts of altruism, particularly in the face of significant personal risk? It is essential to investigate whether there exists a positive influence mechanism linking moral identity, psychological state, and prosocial behaviors.

### Prosocial behavior and its relationship with trait mindfulness

Prosocial behaviors encompass a diverse range of actions aimed at benefiting others, including assisting, consoling, sharing, and collaborating (Davidov et al., 2016). These prosocial behaviors as an important basis for the establishment of positive interpersonal relationships, are positively correlated with individual subjective well-being (Wan et al., 2023), meaning of life (Xie et al., 2023), and creativity (Lin et al., 2023), which may alleviate negative emotions such as loneliness, anxiety, and depression (Inagaki et al., 2022; Zhang et al., 2023). Prosocial tendencies can effectively reflect individual prosocial behavior well, the most common measures of prosocial behaviors assess personal tendencies to exhibit several prosocial behaviors across different contexts and motives (Carlo and Randall, 2002).

For medical students, a specific form of prosocial behavior serves as a driving force behind their pursuit of a medical career and is an essential element of their professionalism (Leffel et al., 2018). It forms the foundational basis for fostering a harmonious doctor-patient relationship, thereby leading to positive outcomes for both healthcare providers and their patients. At present, China has more than 600,000 medical students, and effectively cultivating and mobilizing their prosocial behavior ability is of great significance for improving and enhancing the quality of medical services in the future. However, previous studies have mainly focused on the relationship between prosocial behavior and negative mental health (Arbel et al., 2022; Nagaoka et al., 2022), while research on promoting the level of prosocial behavior is relatively lacking. Even more worrying, a previous review of studies showed that as medical students improved their training and academic performance, their prosocial values declined (Burks and Kobus, 2012). Therefore, it is urgent to further explore the influencing factors and mechanisms of medical students’ social behavior.

The introduction of the Buddhist concept of “mindfulness” by Williams and Kabat-Zinn (2013) into the realm of psychology has offered a novel perspective that broadens the horizons of researchers. Mindfulness entails a non-judgmental and open awareness of one’s present-moment experiences (Brown and Ryan, 2003). This approach to experiencing reality is characterized by direct attention to moment-by-moment occurrences. A growing body of research is now focusing on how mindfulness can enhance human potential and promote positive behaviors, including prosocial behaviors, thereby enabling individuals to flourish at their optimal state (Kuang et al., 2022; Schindler and Pfattheicher, 2023). Mindfulness has been investigated both as an intervention and as a personality trait, and considering the person-situation debate (Fleeson, 2004), both perspectives provide valuable insights into complementary aspects.

Trait mindfulness is defined as one’s predisposition to be mindful in daily life (Kiken et al., 2015). A substantial body of research indicates that trait mindfulness positively affects physical and mental health, promotes empathy (Rezapour-Mirsaleh et al., 2022), fosters a sense of meaning in life (Zhong et al., 2019), and enhances emotional self-regulation (Zhang J. et al., 2022). Importantly, the literature consistently suggests that heightened mindfulness is associated with increased prosocial behavior, as indicated by a meta-analysis revealing a positive correlation between trait mindfulness and prosocial behavior (Lv et al., 2021; Donald et al., 2019). Additionally, some studies propose that the quality of attention directed toward others in social interactions serves as a common intrapsychic factor promoting prosocial behavior (Selwyn, 2014; Singer et al., 2009). Individuals with higher levels of trait mindfulness demonstrate greater awareness of their internal and external environments, which enables them to perceive others’ needs more accurately and engage in prosocial behaviors (Selwyn, 2014; Singer et al., 2009). Prior research has shown that the association between trait mindfulness and prosocial behavior is influenced by several factors. For example, trait mindfulness can indirectly affect prosocial behavior through empathy (Aydin Sünbül, 2021) and self-compassion (Rodríguez-Carvajal et al., 2016). Furthermore, the relationship between trait mindfulness and prosocial behavior can be moderated by various factors such as self-construal and ethical dispositions (Guo et al., 2021; Poulin et al., 2021). This suggests that the effect of trait mindfulness on prosocial behavior is more complex than anticipated. Therefore, this study sought to test the following hypotheses among medical students: (**H1**) Trait mindfulness will significantly predict prosocial behavior.

### Psychological capital and its mediating effect

Psychological Capital (PsyCap) is defined as the positive state of mind exhibited during the growth and development of an individual and comprises four core components: self-efficacy, optimism, hope, and resilience (Luthans et al., 2007a). A substantial body of evidence confirms that the four fundamental components of PsyCap significantly predict levels of prosocial behavior (Malinauskas and Saulius, 2019; Ramolale et al., 2021). Moreover, studies across diverse populations have indicated that PsyCap plays a significant role in fostering the development of prosocial behaviors, with individuals possessing higher levels of PsyCap being more likely to exhibit such behaviors (Liang and Qin, 2022; Zhang S. et al., 2022). In contrast, individuals with low PsyCap exhibit lower levels of prosocial behaviors but higher levels of emotional symptoms and are more predisposed to conduct disorders, hyperactivity, impulsivity, and difficulties in peer interactions (Zhu et al., 2021). In addition, research has also established a positive correlation between trait mindfulness and PsyCap, with PsyCap serving as a significant mediating variable between trait mindfulness and various psychological outcomes, such as subjective wellbeing and emotional exhaustion (Bi and Ye, 2021; Wang and Wu, 2019). Overall, numerous studies have shown a positive association between PsyCap, trait mindfulness, and prosocial behaviors.

The Conservation of Resources (COR) theory can explain the relationships among trait mindfulness, PsyCap, and prosocial behavior. COR theory holds that individuals avert the loss of resources and seek an increase in resources (Hobfoll, 2011). The concept of “resources” forms the foundation of the theory, with both mindfulness and PsyCap being considered as internal resources of the individual (Fisher et al., 2019; Luthans et al., 2007b). COR theory posits that individuals with abundant resources can leverage these existing assets to acquire additional resources (Hobfoll et al., 2018). A heightened level of trait mindfulness signifies a focus on the present moment, unburdened by past or future events, thereby averting ego depletion and ruminative thoughts, which are known to deplete personal resources (Karaoglu and Yalcin, 2020). Consequently, Individuals with a high level of trait mindfulness are more likely to internally perceive their resources as sufficient, with mindfulness inadvertently aiding the accumulation of other personal resources (Brown and Ryan, 2004) and ultimately elevating the level of PsyCap. Furthermore, when resources are plentiful, individuals are encouraged to fully utilize and invest these resources in subsequent activities. Conversely, in situations where resources are relatively scarce, individuals become vulnerable to the risk of resource loss, making it increasingly difficult to invest in and utilize their existing resources (Hobfoll et al., 2018). Since the implementation of prosocial behaviors requires a certain number of personal resources, having a high level of trait mindfulness and PsyCap increases the likelihood that individuals will engage in such behaviors. Therefore, this study posits the following hypotheses: (**H2**) PsyCap plays a mediating role in the relationship between trait mindfulness and prosocial behavior.

### Moral identity and its moderating effect

Moral identity is a crucial psychological mechanism for translating moral cognition into moral behavior (Aquino and Reed Ii, 2002). Medical students are often viewed as the future vanguard of the medical field, highlighting the importance of examining the role that their moral identity plays in fostering prosocial behavior. Moral identity refers to a self-schema organized around moral traits and represents an individual’s recognition of moral characteristics such as caring, loyalty, kindness, fairness, and justice (He and Harris, 2014). This integrates the concepts of self-identity and morality. Numerous studies have consistently demonstrated that moral identity is a significant and positive predictor of prosocial behavior (Gotowiec and van Mastrigt, 2019; Kaur, 2020). Individuals with higher levels of moral identity are more likely to engage in prosocial behaviors. Groups characterized by high levels of moral identity may experience psychological distress when they fail to demonstrate prosocial behavior in situations where it is expected (Winterich et al., 2013).

Moral identity is commonly used as an individual characteristic to delineate variations among individuals. Several studies have indicated that moral identity plays a moderating role in the relationships between several psychological factors and prosocial behavior. For example, the mindfulness-prosociality association among undergraduate students with higher or lower levels of ethical dispositions showed that trait mindfulness was more strongly associated with prosociality among lower relative to higher ethical individuals (Guo et al., 2021). However, another study suggested that compared to individuals with low moral identity, those with high moral identity exhibit a stronger correlation between moral elevation and prosocial behavior (Wan et al., 2023). Individuals with higher levels of moral identity exhibit a strong desire to align their moral identity with their actual behavior. They place greater emphasis on the significance of moral character and tend to uphold their internal moral standards, which reduces the likelihood of moral disengagement and enhances the probability of engaging in prosocial behavior (Zhao and Qu, 2022). Individuals with low moral identity have diminished moral self-control and tend to disregard the effects of internal moral traits. Consequently, moral decision-making is more contingent on external perceptions (Moore et al., 2019). These above findings suggest that the role of moral identity in the relationship between mindfulness and prosocial behavior is worthy of further exploration.

In this study, we propose that moral identity, as a key trait reflecting individual differences, may act as a moderator in the relationship between trait mindfulness, PsyCap, and prosocial behavior. The effects of trait mindfulness and PsyCap on promoting prosocial behavior may be amplified in groups with higher levels of moral identity. In other words, the impact of trait mindfulness and PsyCap on prosocial behavior may depend on the level of moral identity. Individuals with higher levels of moral identity are more likely to benefit from elevated levels of trait mindfulness and PsyCap. In this study, the following assumptions were made: (**H3a**) Moral identity moderates the relationship between trait mindfulness and prosocial behavior; (**H3b**) Moral identity moderates the relationship between PsyCap and prosocial behavior.

**Figure 1:**
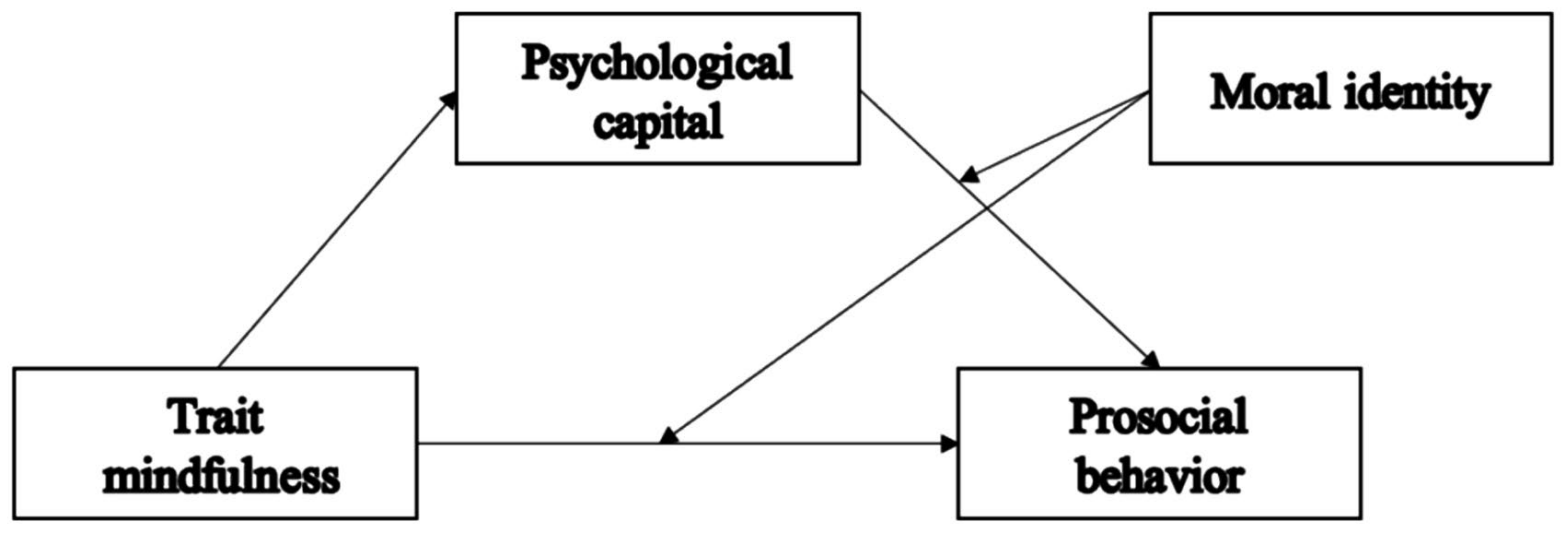
A hypothetical model of trait mindfulness, prosocial behavior, psychological capital, and moral identity among medical students. Mediating variable, PsyCap; Moderating variable, moral identity.

In summary, we propose a moderated mediation effect in this study, as illustrated in Figure 1.

## Methods

### Sample and procedure

A cross-sectional survey was conducted between July and October 2023, using cluster stratified random sampling to recruit medical students from four medical colleges in Jiangsu Province, such as clinical medicine, pharmacy, nursing, traditional Chinese medicine, medical imaging, and rehabilitation therapy. Firstly, we stratified medical students by grade, including four grades from freshman to senior. Secondly, at least one complete class from each grade in different majors at each medical school was randomly selected by cluster sampling.

To optimize data collection efficiency, data gathering was scheduled during times when students were primarily gathered in the classroom, such as during morning or evening self-study sessions and class meetings. The questionnaire collected data in an anonymous and self-reported way. The investigator and the class counselor explained the purpose of the survey to the participants and assured the collected data would be used solely for scientific research, ensuring the protection of their privacy. We prepared the questionnaire in advance on the “Questionnaire Star” online platform, and all participants completed the questionnaire by scanning a QR code on site and then filling it out.

According to the criterion (the number of items increased by 10–20 times and expanded by at least 10%) by Kendall (Lu and Fang, 2002), the total number of items in the four scales in this study was 105, so the sample size was no less than 1,155. The inclusion criteria required participants to be full-time students who signed an informed consent form online to voluntarily participate in the study. Exclusion criteria include short completion time, response regularity, and suspected extreme values.

### Measurements

*Prosocial Tendencies Measurement Scale (PTM)*. This study employed the PTM originally developed by Carlo and Randall (2002) to evaluate prosocial behavior among medical students. This 26-item scale covers six dimensions: openness, anonymity, altruism, compliance, emotion, and urgency. Responses were recorded on a five-point Likert scale ranging from 1 (strongly disagree) to 5 (strongly agree). The Chinese version has shown good reliability among Chinese university students (Zhang S. et al., 2022). The PTM displayed a high level of internal consistency reliability in this study, with a Cronbach’s *α* coefficient of 0.949.

*Five-Facet Mindfulness Questionnaire (FFMQ)*. This study used the FFMQ, initially developed by Baer et al. (2006), to assess mindfulness among medical students. It consists of 39 items evaluating five dimensions of mindfulness: observation, description, conscious movement, nonjudgment, and nonreaction. Responses were recorded on a five-point Likert scale, with higher scores indicating greater mindfulness. The FFMQ demonstrated high reliability and validity among Chinese youth populations (Li and Mu, 2020) and exhibited strong internal consistency reliability in this study, with a Cronbach’s *α* coefficient of 0.910.

*Psychological Capital Questionnaire (PCQ-24)*. This study used the PCQ-24 developed by Luthans et al. (2007b) to measure PsyCap. Comprising 24 items across four dimensions, the PCQ-24 asks respondents to rate each item on a six-point Likert scale, with higher scores indicating elevated PsyCap levels. The Chinese version has shown good reliability among Chinese university students (Xu et al., 2022) and demonstrated strong internal consistency reliability in the current study, with a Cronbach’s *α* coefficient of 0.928 for the total scale.

*Moral Identity Scale (MIS)*. This study employed the Moral Identity Scale (MIS), initially developed by Aquino and Reed Ii (2002), to evaluate the moral identity of medical students. The scale consists of 16 items and uses a five-point Likert score method, with higher scores indicating greater levels of moral identification within an individual. The Chinese version has shown good reliability among young Chinese (Wan et al., 2023). In this study, the MIS demonstrated strong internal consistency reliability, with a Cronbach’s *α* coefficient of 0.911.

### Data analysis

The SPSS version 25.0 was employed to implement descriptive statistics and correlation analysis with *p* < 0.05 (two-tailed test) as statically significant. The impact of various demographic factors on prosocial behavior was assessed using independent sample *t*-tests or one-way ANOVA. Pearson’s correlation analysis was conducted to examine the relationships between trait mindfulness, PsyCap, moral identity, and prosocial behavior. All models were adjusted for sociodemographic factors such as sex, age, and ethnic minority status. Following the standardization of all data and utilizing 5,000 bootstrap samples, the mediating effect of PsyCap and the moderation of this mediation by moral identity were analyzed using Models 4 and 15 of PROCESS v3.4 (Hayes, 2018), respectively. Statistical significance was determined based on confidence intervals excluding zero.

### Ethical approval

The ethics committee of Xuzhou Medical University approved this study. All procedures were carried out in accordance with relevant ethical guidelines and regulations. All participants signed an online informed consent form and voluntarily participated in this study.

## Results

### Common method bias

Data collection for this study involved the use of self-report questionnaires, which may have introduced common method bias. Harman’s single-factor test was applied to assess the potential impact of common method bias on the study results (Podsakoff et al., 2003). The analysis revealed that the explanatory power of the first factor was 22.44%, which is below the 40% threshold. This indicates that there was no significant common method bias affecting the study results.

### Descriptive statistics and correlation analysis

A total of 2,500 students participated in the survey, of which 2,285 valid samples were collected with a valid recovery rate of 91.4%. The sample comprised 689 males (30.15%) and 1,596 females (69.85%). Their mean age was 19.84 ± 1.53 years, with proportions of freshmen, sophomores, juniors, and seniors at 24.51, 19.43, 26.91, and 29.15%, respectively. Most students (59.47%) were from rural areas, and 42.28% were only child. A small proportion (8.67%) was from single-parent families. Furthermore, the majority were ethnic Han (94.84%), only 5.16% were ethnic minority, and 2.71% held religious beliefs among them. Notably, there were no significant differences in the prosocial behavior scores of medical students among the various subgroups categorized by grade, home location, only child status, single-parent family, or religious beliefs (*p* > 0.05) (Table 1).

**Table 1:** Sociodemographic characteristics and differences of medical students’ prosocial behavior (*N* = 2,285).

Variables	*N* (%)	M ± SD	*t/F*	*p*
Gender				
Male	689 (30.15)	96.53 ± 15.12	2.541	0.011
Female	1,596 (69.85)	94.82 ± 13.99		
Ages (years)				
<20	1,004 (43.94)	96.15 ± 14.01	2.170	0.030
≥20	1,281 (56.06)	94.69 ± 14.60		
Grade				
Freshmen	560 (24.51)	96.20 ± 13.98	2.255	0.080
Sophomores	444 (19.43)	96.08 ± 14.05		
Juniors	615 (26.91)	95.10 ± 14.23		
Seniors	666 (29.15)	94.32 ± 14.93		
Home location				
Rural areas	1,359 (59.47)	95.65 ± 14.82	1.311	0.190
Urban areas	926 (40.53%)	94.86 ± 13.65		
Only child				
Yes	966 (42.28%)	95.09 ± 14.80	−0.699	0.484
No	1,319 (57.72)	95.51 ± 14.02		
Single-parent family				
Yes	198 (8.67)	94.85 ± 14.18	−0.497	0.619
No	2,087 (91.33)	95.38 ± 14.38		
Ethnic minority				
Yes	118 (5.16)	93.08 ± 11.77	−2.110	0.037
No	2,167 (94.84)	95.46 ± 14.48		
Religious belief				
Yes	62 (2.71)	95.03 ± 15.52	−0.167	0.867
No	2,223 (97.29)	95.34 ± 14.33		

M, mean; SD, standard deviation.

In the correlation analysis, trait mindfulness was positively correlated with PsyCap (*r* = 0.592, *p* < 0.01) and prosocial behavior (*r* = 0.293, *p* < 0.01). Additionally, PsyCap was positively correlated with prosocial behavior (*r* = 0.444, *p* < 0.01). Furthermore, moral identity was positively correlated with trait mindfulness (*r* = 0.289, *p* < 0.01), PsyCap (*r* = 0.559, *p* < 0.01), and prosocial behavior (*r* = 0.528, *p* < 0.01).

### The mediating role of psychological capital

**Figure 2:**
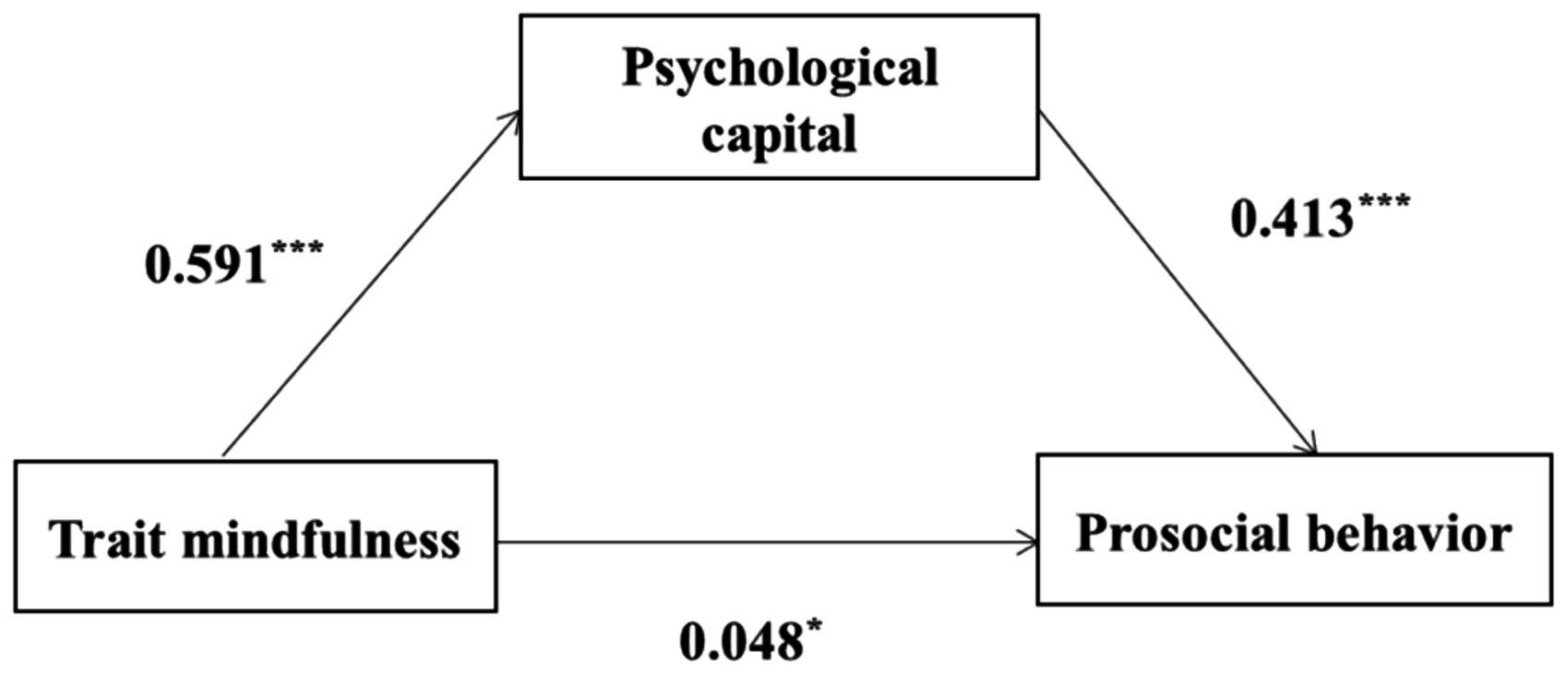
Psychological capital plays a mediating role between trait mindfulness and prosocial behavior. ^***^p < 0.001; ^*^p < 0.05.

Process Model 4 was employed to examine the mediating role of PsyCap on trait mindfulness and prosocial behavior. Table 2 lists the results of the regression tests. Trait mindfulness had a significant positive effect on prosocial behavior. After incorporating PsyCap as the mediating variable in the equation, the positive predictive effect of trait mindfulness on prosocial behavior remained significant. Furthermore, the positive predictive effect of trait mindfulness on PsyCap was significant, and PsyCap exhibited a significant positive predictive effect on prosocial behavior. Hence, PsyCap was observed to play a partial mediating role between trait mindfulness and prosocial behaviors (*β* = 0.413, 95%CI [0.368, 0.459]). This model is illustrated in Figure 2.

**Table 2:** The mediating role of PsyCap between trait mindfulness and prosocial behavior.

Variables	Model 1 (Prosocial behavior)			Model 2 (PsyCap)			Model 3 (Prosocial behavior)		
	*β*	*t*	95% CI	*β*	*t*	95% CI	*β*	*t*	95% CI
Trait mindfulness	0.292	14.640^***^	[0.253, 0.332]	0.591	35.100^***^	[0.521, 0.587]	0.048	2.072^*^	[0.003, 0.094]
PsyCap							0.413	17.746^***^	[0.368, 0.459]
*R*^2^	0.092			0.355			0.202		
*F*	57.639			313.362			115.440		

PsyCap, psychological capital.

^***^p < 0.001, ^*^p < 0.05.

### The moderating effect of moral identity

**Figure 3:**
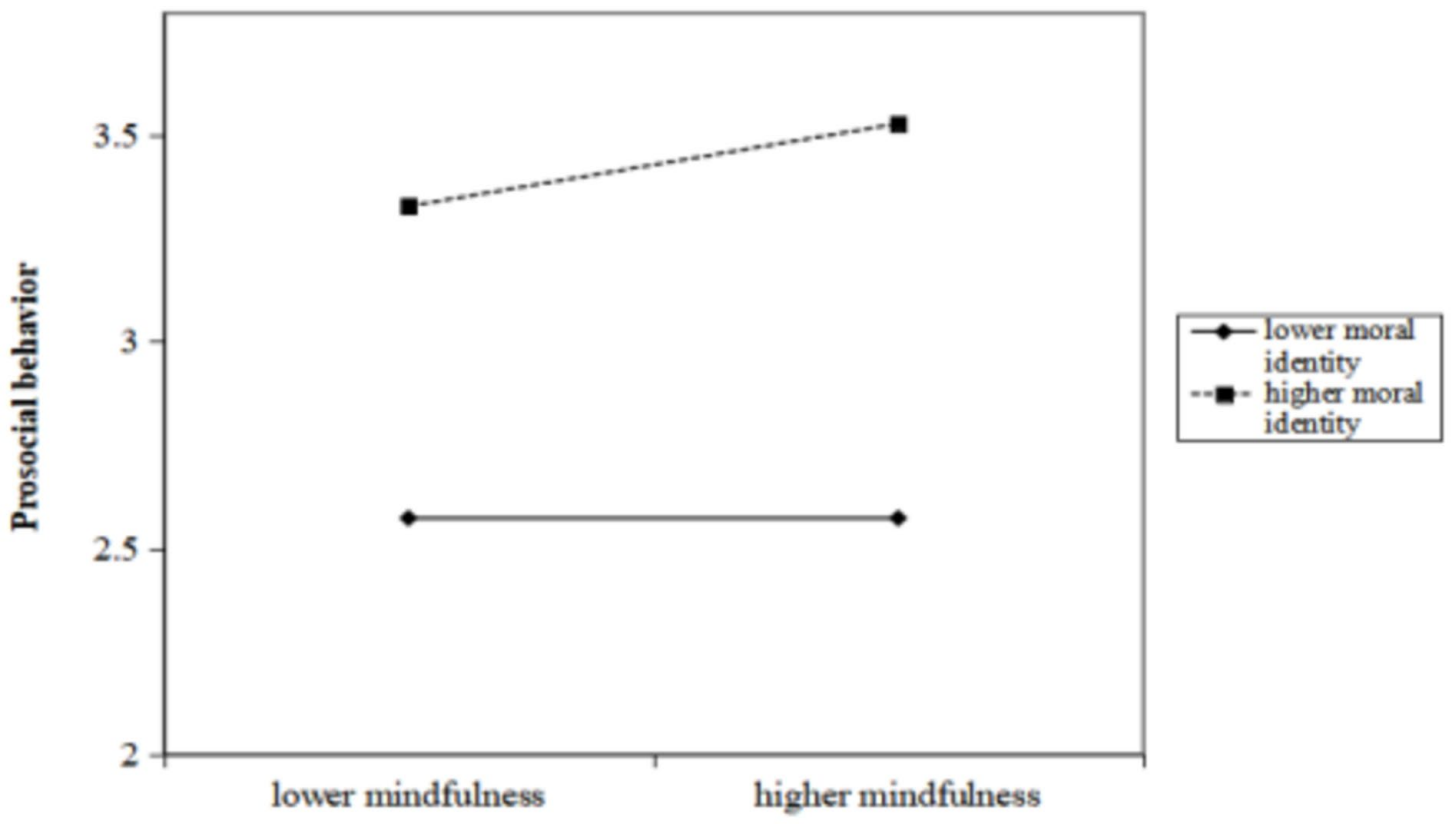
Interaction effect of trait mindfulness and moral identity on prosocial behavior. High and low levels of trait mindfulness and moral identity represent one standard deviation above and below the mean, respectively.

**Figure 4:**
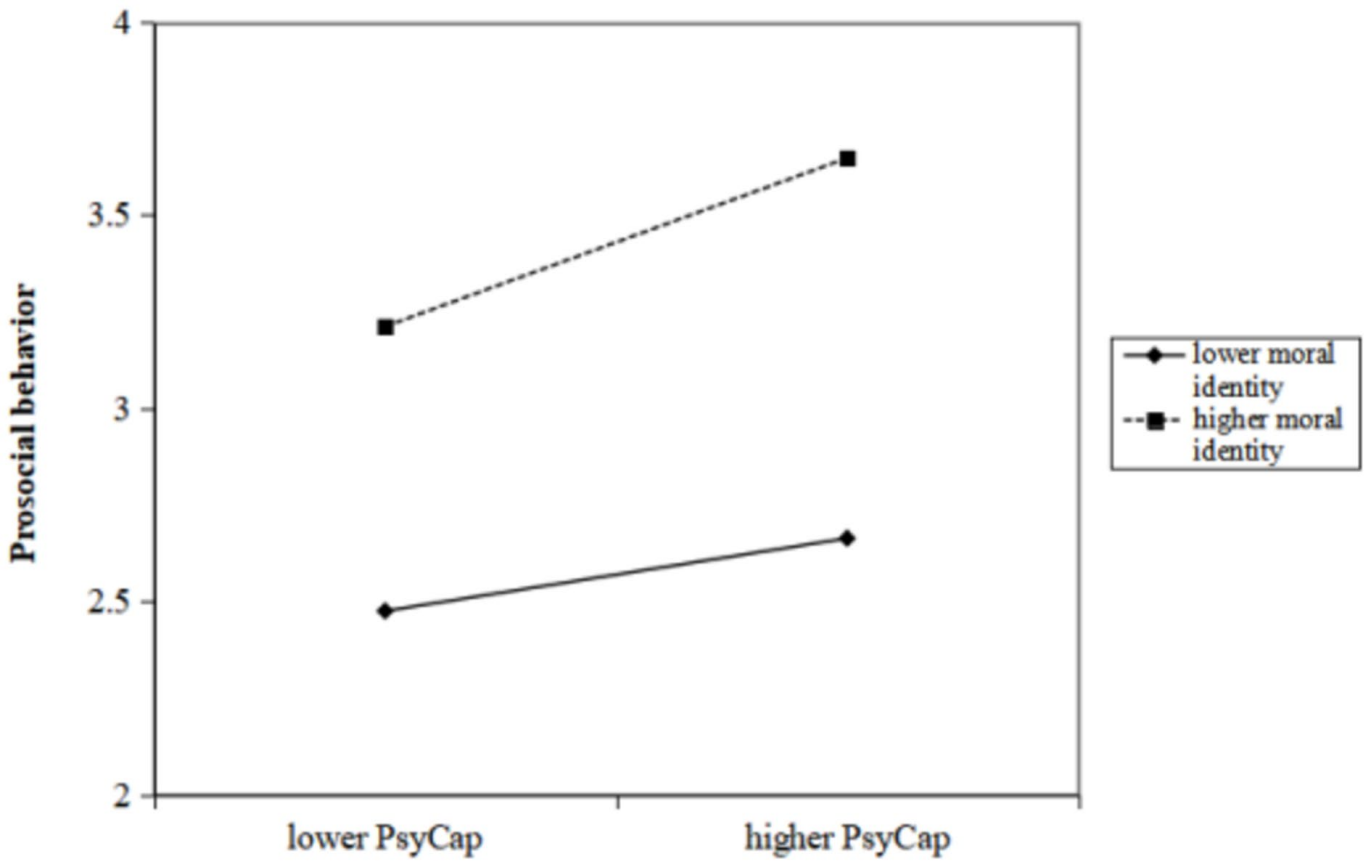
Interaction effect of PsyCap and moral identity on prosocial behavior. High and low levels of PsyCap and moral identity represent one standard deviation above and below the mean, respectively.

Having identified the mediating effect of PsyCap on the relationship between trait mindfulness and prosocial behavior, we sought to explore whether moral identity moderates this relationship. The results revealed that the interaction of trait mindfulness with moral identity significantly predicted prosocial behavior (*β* = 0.049, 95%CI [0.011, 0.087]), and similarly, the interaction of PsyCap with moral identity significantly predicted prosocial behavior (*β* = 0.062, 95%CI [0.032, 0.092]) (Table 3). Following this, we conducted a simple slope analysis to predict the relationship between trait mindfulness and prosocial behavior as well as between PsyCap and prosocial behavior separately for participants with high and low levels of moral identity. Figure 3 illustrates that the slope of the association between trait mindfulness and prosocial behavior was strong for participants with high moral identity (*β*_high moral identity_ = 0.096, *t* = 3.617, *p* < 0.001), while it did not reach a significant level for participants with low moral identity (*β*_low moral identity_ = −0.003, *t* = −0.097, *p* > 0.05). Furthermore, as depicted in Figure 4, the slope of the association between PsyCap and prosocial behavior was relatively weak for participants with low moral identity (*β*_low moral identity_ = 0.094, *t* = 3.191, *p* < 0.01), whereas the slope was relatively strong when the moral identity of participants was high (*β*_high moral identity_ = 0.218, *t* = 7.644, *p* < 0.001). The direct and indirect effects of trait mindfulness on prosocial behavior at different levels of moral identity are presented in Table 4.

**Table 3:** Testing the moderating mediation effect of moral identity.

Predictors	Model 1 (PsyCap)			Model 2 (Prosocial behavior)		
	*β*	*t*	95% CI	*β*	*t*	95% CI
Trait mindfulness	0.591	35.100^***^	[0.558, 0.624]	0.046	2.108^*^	[0.003, 0.089]
PsyCap				0.156	6.311^***^	[0.107, 0.204]
Moral identity				0.430	20.519^***^	[0.389, 0.471]
Interaction term 1				0.049	2.545^*^	[0.011, 0.087]
Interaction term 2				0.062	4.110^***^	[0.032, 0.092]
*R*^2^	0.355			0.333		
*F*	313.362			141.809		

PsyCap, psychological capital; Interaction term 1, Trait mindfulness × Moral identity; Interaction term 2, PsyCap × Moral identity.

^***^p < 0.001, ^*^p < 0.05.

**Table 4:** Direct and indirect effects at different levels of moral identity.

	Moral identity	Effect	SE	95% bootstrap CI
Direct effect	M − 1SD	−0.003	0.032	[−0.066, 0.059]
	M	0.046	0.022	[0.003, 0.089]
	M + 1SD	0.096	0.026	[0.044, 0.147]
Indirect effect	M − 1SD	0.055	0.025	[0.008, 0.106]
	M	0.092	0.019	[0.058, 0.130]
	M + 1SD	0.129	0.024	[0.082, 0.177]

M, mean; SD, standard deviation.

## Discussion

The present study identified significant differences in prosocial behavior among medical students based on gender, age, and ethnicity. Notably, our findings indicate that females exhibit lower levels of prosocial behavior compared to males, which contradicts the research that documents higher prosocial behavior among girls (Carlo et al., 2015). However, Nielson et al. suggest that gender differences in prosocial behavior are not absolute, they may be influenced by situational and cultural contexts (Nielson et al., 2020). Furthermore, the observed gender differences in prosocial behavior may be partly attributed to methodological variations in how prosocial behavior is assessed. Additionally, this study reveals that older medical students demonstrate lower levels of prosocial behavior than their younger counterparts. This decline may be attributed to the increasing academic and workload demands faced by students as they advance in their education, which could lead to a decrease in prosocial behavior (Burks and Kobus, 2012). Our study also found that ethnic minority students exhibited lower levels of prosocial behavior compared to Han Chinese students. This disparity may stem from difficulties ethnic minorities face in integrating with peers, often due to language barriers and differing cultural practices. These results underscore the need to focus on fostering prosocial behavior among female, senior, and ethnic minority medical students.

This study investigated the effects of trait mindfulness on prosocial behavior and its underlying mechanisms by constructing a model of mediated moderation among medical students. This study demonstrated that there is a positive association between trait mindfulness and prosocial behavior among medical students, and trait mindfulness indirectly affects prosocial behavior through PsyCap, as well as the mediation is moderated by moral identity. All the proposed assumptions are supported by the findings.

First, the results revealed a positive correlation between trait mindfulness and prosocial behavior, indicating that individuals with higher levels of trait mindfulness are more inclined to engage in prosocial behavior, thereby supporting Hypothesis 1. Trait mindfulness is a personality attribute cultivated through mindfulness practice (Kiken et al., 2015), and individuals can increase trait mindfulness by repeatedly evoking a state of mindfulness across meditation sessions (Davidson, 2010). Mindfulness as a distinct internal resource differs from traditional internal resources such as PsyCap. It is more closely related to how individuals utilize their attentional resources (Good et al., 2016). Mindfulness emphasizes the acceptance of everything as it is and enables individuals to experience greater freedom from life and a deeper sense of acceptance (Baljinder et al., 2016). Consequently, this enhances the likelihood of individuals responding beneficially to the needs of others, including displaying more significant prosocial behaviors toward out-group members (Clobert et al., 2015). Existing research has shown that mindfulness practice leads to a reduction in the amygdala response, which is associated with distress, and an increase in the anterior insular response, which is involved in emotional production and regulation. These changes in brain activity facilitate heightened awareness of others’ suffering and increase the propensity to engage in prosocial behaviors (Weng et al., 2013). Compared to students majoring in other fields, the development of prosocial behavior of medical students will play a more important role in their interactions with patients, the establishment of a good doctor-patient relationship, and the medical atmosphere. Therefore, we suggest that it is necessary to introduce mindfulness training into medical education to promote the development of prosocial behavior by enhancing the trait mindfulness of medical students.

Second, the present study finds that PsyCap plays a mediating role in the relationship between trait mindfulness and prosocial behavior, thus confirming hypothesis 2, which is an important finding of this study. No previous research has explored the relationship between trait mindfulness, PsyCap, and prosocial behavior jointly among medical students. This study found that trait mindfulness can predict psychological capital, which is consistent with previous studies underscoring the influential role of trait mindfulness in bolstering an individual’s PsyCap (Bi and Ye, 2021). First of all, mindfulness as a personal resource can assist individuals in recognizing their current resource levels, reducing their reliance on external resources, and expanding their awareness of alternative resources (Kroon et al., 2015), ultimately leading to an enhancement in PsyCap. In addition, according to COR theory, Individuals are driven to allocate and utilize resources, and having an initial surplus of resources motivates them to fully leverage and invest in their existing resources, as well as dedicate them to subsequent activities (Hobfoll, 2011). Consequently, an individual’s assessment of his/her resources directly affects his/her subsequent behavior. Individuals with ample PsyCap often exhibit a positive outlook on life events, attributing distress to external factors and displaying a greater inclination to offer assistance, even to strangers, compared to those with lower levels of PsyCap (Zhang S. et al., 2022). Conversely, following the first principle of COR theory, which emphasizes that resource loss holds greater significance than resource gain (Hobfoll et al., 2018), individuals with low trait mindfulness, easily caught up in ego depletion or rumination, feel potentially threatened by resources, leading to reluctance to invest and utilize available resources. Therefore, they are more inclined to adopt protective measures to prevent further depletion, inadvertently resulting in a decline in prosocial behaviors. These findings have significant practical implications for enhancing prosocial behaviors among medical students. While emphasizing the importance of directing medical students’ attention to the present moment to foster trait mindfulness, equal emphasis should be placed on fostering positive, optimistic, and resilient attitudes to enhance their PsyCap, thereby promoting prosocial behavior.

Third, the results revealed that moral identity plays a moderating role in the relationship between trait mindfulness and prosocial behavior, confirming Hypothesis 3a. As the level of moral identity increases, the relationship between trait mindfulness and prosocial behavior becomes stronger. When medical students have high levels of trait mindfulness, high levels of moral identity will lead to more prosocial behavior. This suggests that alongside using mindfulness training to enhance medical students’ prosocial behaviors, it is equally important to elevate their moral identity to further promote the development of prosocial behavior. Within Buddhist traditions, mindfulness embodies a more comprehensive and profound concept than presently acknowledged and applied in psychology (Kang and Whittingham, 2010). Some scholars argue that mindfulness inherently includes ethical speech and actions as part of a complex set of interrelated processes (Greenberg and Mitra, 2015). However, contemporary secular mindfulness, as widely defined, often omits the formal consideration of morality and ethics and instead emphasizes the non-judgmental awareness of present events and experiences. There is an ongoing debate about whether ethics should be added to the practice of mindfulness. Some scholars argue that merely cultivating mindfulness is insufficient to foster prosocial engagement (Monteiro et al., 2015). For example, a study involving German students revealed that the non-judgmental aspect of mindfulness can attenuate moral responses, such as feelings of guilt (Schindler et al., 2019). These findings highlight the complementary and inseparable nature of moral identity and positive thinking. In this study, a moderate-to-high level of moral identity is a critical prerequisite for trait mindfulness to be effective in promoting prosocial behavior. For medical students with low moral identity, it is difficult to improve their prosocial behavior level by increasing their trait mindfulness. However, another study demonstrated that the link between trait mindfulness and prosocial behavior is not strong among highly ethical individuals (Guo et al., 2021). This conclusion may be due to utilizing 121 scale items with a sample size of only 709, which significantly is less than the recommended 10–20 times sampling requirement (Lu and Fang, 2002). Furthermore, the study did not report on the issue of common methodological bias, which may have led to findings that contrast with those of the current study, and is worth exploring further in future studies. Our study emphasizes that while striving to foster trait mindfulness in medical students and enhance their PsyCap, we should pay equal attention to nurturing their moral identities.

Finally, this study’s findings demonstrate that moral identity plays a moderating role in the relationship between PsyCap and prosocial behavior, thus confirming Hypothesis 3b. PsyCap emerged as a significant predictor of prosocial behavior, regardless of the level of moral identity; however, its predictive capacity for prosocial behavior strengthened as moral identity reached higher levels. This trend can be explained by moral identity as a self-regulatory system that motivates moral action. Individuals with a heightened moral identity demonstrate a broader scope of moral concerns and are predisposed to engage in prosocial behaviors, such as volunteering activities (Patrick et al., 2018). Those with a strong moral identity, identifying themselves as caring, friendly, and amicable, often exhibit elevated moral self-control and strive to align their behavior with their ethical standards. Research has shown that individuals’ distinct moral identity standards directly influence the extent of psychological resource loss and subsequent decision-making processes when reflecting on and contemplating unethical events (Greenbaum et al., 2014). Therefore, PsyCap functions as a psychological resource, and not all individuals readily engage in prosocial behaviors despite possessing sufficient PsyCap. Higher levels of moral identity appear to facilitate the association between PsyCap and engagement in prosocial behaviors. Individuals with elevated levels of both PsyCap and moral identity demonstrate a heightened ability to recognize the needs of others and, consequently, engage in prosocial behavior. Conversely, individuals with lower levels of moral identity, due to their diminished focus on conforming to moral norms and lower moral self-control, may exhibit a comparatively reduced willingness to engage in prosocial behavior, even with sufficient PsyCap. This underscores the stronger association between PsyCap and prosocial behaviors among individuals with a high moral identity than among those with a low moral identity. Consequently, while focusing on developing the PsyCap of medical students, it is imperative to direct attention toward enhancing their moral identity to effectively promote their engagement in prosocial behavior.

Several limitations of this study should be acknowledged. First, the cross-sectional design did not provide a causal relationship between trait mindfulness, PsyCap, moral identity, and prosocial behavior. Future longitudinal or experimental studies are needed to begin exploring possible causal links between variables. Second, the sample was limited to medical students from Jiangsu Province, China and the context of all participants was identified as the eastern culture. It is not ascertained whether the findings can be generalized to students from other provinces or students from other cultures. Future studies should recruit a larger and more diverse sample to ensure that the findings can represent the entire situation. Third, this study relies primarily on self-reported prosocial behavior. Subsequent research efforts could explore the relationship between trait mindfulness and a variety of different types of prosocial behavior, thus providing a more comprehensive understanding of the topic.

## Conclusion

In conclusion, trait mindfulness has a positive correlation with prosocial behavior, medical students with higher levels of trait mindfulness are more inclined to engage in prosocial behavior. PsyCap mediated the effect of trait mindfulness on prosocial behavior, with the moral identity moderating role between trait mindfulness and prosocial behavior, PsyCap, and prosocial behavior. Our study provides evidence for the development of mental health courses tailored to medical students and interventions aimed at enhancing prosocial behaviors. Specifically, efforts focus on cultivating trait mindfulness and PsyCap can enhance medical students’ prosocial behaviors; Initiatives aimed at promoting moral identity among medical students could further facilitate the effect of trait mindfulness and PsyCap on prosocial behavior.

The theoretical implications of this study are threefold. First, the findings highlight the importance of the interconnected relationships between trait mindfulness, PsyCap, and prosocial behavior, thus enhancing our understanding of these dynamics specifically among medical students—a topic that has not been previously explored in research. Second, this study contributes to the literature on the antecedent variables influencing prosocial behavior, grounded in COR theory, addressing gaps in previous research that have been limited in previous research on promoting prosocial behavior. Finally, drawing on COR theory, this study elucidates the mechanisms by which trait mindfulness and PsyCap influence prosocial behavior, particularly emphasizing the unique role of trait mindfulness as an individual resource that facilitates the acquisition of additional resources and fosters prosocial behavior. This enriches the conceptual framework of resources within COR theory.

## Data Availability

The original contributions presented in the study are included in the article/supplementary material, further inquiries can be directed to the corresponding author.
